# Temperature-Responsive Magnetic Nanoparticles for Bioanalysis of Lysozyme in Urine Samples

**DOI:** 10.3390/nano11113015

**Published:** 2021-11-10

**Authors:** Marwa A. Ahmed, Júlia Erdőssy, Viola Horvath

**Affiliations:** 1 Department of Inorganic and Analytical Chemistry, Faculty of Chemical Technology and Biotechnology, Budapest University of Technology and Economics, H-1111 Budapest, Hungary; marwaaa@a-edu.suez.edu.eg (M.A.A.); julia.erdossy@gmail.com (J.E.); 2 Department of Chemistry, Faculty of Science, Arish University, El-Arish 45511, Egypt; 3 MTA-BME Computation Driven Chemistry Research Group, H-1111 Budapest, Hungary

**Keywords:** thermoresponsive polymer, magnetic nanoparticle, lysozyme, bioanalytical method validation

## Abstract

Highly selective multifunctional magnetic nanoparticles containing a thermoresponsive polymer shell were developed and used in the sample pretreatment of urine for the assessment of lysozymuria in leukemia patients. Crosslinked poly(N-isopropylacrylamide-co-acrylic acid-co-N-tert-butylacrylamide) was grown onto silica-coated magnetic nanoparticles by reversible addition fragmentation chain transfer (RAFT) polymerization. The lysozyme binding property of the nanoparticles was investigated as a function of time, protein concentration, pH, ionic strength and temperature and their selectivity was assessed against other proteins. High-abundant proteins, like human serum albumin and γ-globulins did not interfere with the binding of lysozyme even at elevated concentrations characteristic of proteinuria. A sample cleanup procedure for urine samples has been developed utilizing the thermocontrollable protein binding ability of the nanoparticles. Method validation was carried out according to current bioanalytical method validation guidelines. The method was highly selective, and the calibration was linear in the 25 to 1000 µg/mL concentration range, relevant in the diagnosis of monocytic and myelomonocytic leukemia. Intra- and inter-day precision values ranged from 2.24 to 8.20% and 1.08 to 5.04%, respectively. Intra-day accuracies were between 89.9 and 117.6%, while inter-day accuracies were in the 88.8 to 111.0% range. The average recovery was 94.1 ± 8.1%. Analysis of unknown urine samples in comparison with a well-established reference method revealed very good correlation between the results, indicating that the new nanoparticle-based method has high potential in the diagnosis of lysozymuria.

## 1. Introduction

Endowing nanoparticles with different functionalities will multiply the attractive properties stemming from their nanoscale dimensions, and widely extend their scope of application in biomedicine [1], catalysis [2], electronics [3] and analytical chemistry [4,5]. Combined use of polymeric materials with magnetic nanoparticles (MNPs) are intensively explored in biomedical applications like imaging, drug delivery and tissue engineering [6] and in bioanalysis [7]. In the latter field the high specific surface area of the nanoparticles combined with the ease of separation due to their superparamagnetic properties makes them excellent candidates as sorbent materials for the separation and enrichment of proteins [8,9]. The carefully chosen polymer shell can serve multiple purposes. First of all, it increases stability and prevents aggregation of the nanoparticles [10]. Moreover, it can lend selectivity to the sorbent material as well as a high affinity towards a predefined target protein. Stimuli-responsive polymers can add further unique properties to these multifunctional nanoparticles like light-, temperature- or pH-controlled binding and release of the target analyte [11].

Two fundamental approaches can impart selectivity and high affinity to the polymer: (i) molecular imprinting [12] and (ii) the use of monomers with carefully selected functional groups in optimized composition [13]. There are many publications on the design and synthesis of molecularly imprinted polymer coated MNPs for the extraction of biomacromolecules from biological samples [14]. Indeed, there exist reports whereby selectivity in a linear polymer [15], or in a crosslinked polymer nanoparticle [16,17], to a defined target protein is achieved by using monomers bearing functional groups that complement protein domains. The optimal polymer is usually selected from a combinatorial library of polymers with diverse functional groups and ratios of the monomers. Up to now the latter approach has only been applied to synthesize core–shell magnetic nanoparticles for protein recognition by Zhang et al. [18].

Lysozyme is a ubiquitous hydrolytic enzyme found in many living organisms. It is capable of cleaving peptidoglycans in the cell membrane of Gram positive bacteria, therefore providing antimicrobial protection [19]. Lysozyme is widely distributed through the human body but its concentration in different body fluids shows a great variation [20]. It is extremely abundant in secretions such as tears, breast milk, gastric juice, pus and nasal mucus and it can also be found in serum, saliva, and sperm. Urine, bile and cerebrospinal fluid contain only minute amounts of lysozyme [20]. The normal range in serum is between 6–14 µg/mL, while in urine, concentrations less than 1 µg/mL are considered normal [20,21,22], although it has to be noted that different reference intervals were obtained using various analytical methods [23]. Elevated serum levels of lysozyme might be associated with pathological conditions like rheumatoid arthritis [23] and Crohn’s disease [23]. Increased urine and serum concentrations are observed in urogenital tumors [22] and renal dysfunction [21] and extremely high serum (5–230 µg/mL) and urinary (up to 1600 µg/mL) concentrations are observed in monocytic and myelomonocytic leukemia [21,24]. Urinary lysozyme levels have differential diagnostic value in monocytic or myelomonocytic leukemia and can be used to follow the patient’s response to cancer therapy.

Traditional methods for the quantitation of lysozyme in clinical samples are based on enzyme activity measurements, originally developed by Smolelis [25]. These are based on the lytic action of lysozyme on the cell walls of *Micrococcus lysodeikticus* and the clearing rate of the originally turbid bacterial suspension is measured. Using the same principle, an agar plate (lysoplate) method with a much wider concentration range has been elaborated by Osserman and Lawson and has seen widespread use in clinical laboratories [24]. These methods, however, depend on the activity of lysozyme which is not easily correlated to its concentration. Radio- [26], enzyme- [23], luminescent- [27] and microparticle-based nephelometric [28] immunoassays for lysozyme provide extremely high sensitivity for the analysis of clinical samples. Other well-established techniques like liquid chromatography [29] and liquid chromatography coupled to mass spectrometry [30] are used mainly in food analysis.

Emerging techniques for lysozyme measurement in biological samples include antibody [31,32,33], aptamer [34], MIP [35] or nanoparticle-based [36] biosensors and the use of core–shell polymer NPs as selective extraction sorbents in the sample cleanup process. Most of the newly developed lysozyme nanoadsorbents use a molecular imprinting strategy [37,38,39,40,41,42,43,44,45,46] and only one of them relies on a polymeric material [18] with optimized composition of special functional monomers to achieve selectivity. The majority of the works focus on the preparation and characterization of the adsorbent particles but, unfortunately, the demonstration of their practical applicability through validated method development has received very little attention up to now. Jing et al. reported a validated clinical method using MIP MNPs for the sample cleanup of highly diluted urine samples coupled with chemiluminescence detection [47], however, the selectivity of the NPs against interfering proteins was quite low [43].

In this paper we propose novel multifunctional hydrogel coated magnetic nanoparticles as highly selective sample enrichment devices for the clinical measurement of lysozyme in urine samples. A polymer layer bearing appropriate functionalities in an optimized composition [16] is coated onto preformed RAFT agent-functionalized MNPs using an initiator system that we earlier optimized [48]. Owing to their thermoresponsive nature, the obtained nanoparticles (Lys-PMNPs) can extract lysozyme with very high selectivity from urine at room temperature, and the bound protein can be released into buffer at low temperature and quantitated. We were also motivated to elaborate a bioanalytical method whereby different experimental variables of the selective extraction process were thoroughly optimized. Finally, the method was validated according to bioanalytical method validation guidelines [49], thereby fostering the application of protein-selective MNPs from research stage to routine analysis. In Table S1 the performance of the Lys-PMNPs is compared with other lysozyme-selective nanoparticles described in the literature.

## 2. Materials and Methods

### 2.1. Materials

All reagents used were at least of analytical grade. Ferric chloride (FeCl_3_·6H_2_O), polyethylene glycol 4000 (PEG 4000), polyethylene glycol 400 (PEG 400), ethylene glycol (EG), anhydrous sodium acetate (NaOAc), 3-aminopropyltrimethoxysilane (APTMOS), N-isopropylacrylamide (NIPAm), acrylic acid (AAc), N,N-methylene bisacrylamide (BIS), ammonium persulfate (APS), sodium dodecyl sulfate (SDS), sodium bisulfite (NaHSO_3_), N,N,N’,N’-tetramethylenediamine (TEMED), trifluoroacetic acid (TFA), 4-cyano-4-(phenylcarbonothioylthio)pentanoic acid (RAFT), lysozyme from chicken egg white (Lys; MW 14.3 kDa, pI 11.35), avidin (MW 68 kDa, pI 10.5), cytochrome c (cyt c; MW 12.3 kDa, pI 9.6), bovine serum albumin (BSA; MW 66.5 kDa, pI 4.7), albumin from chicken egg (MW 44.3 kDa, pI 4.54), human serum albumin (HSA¸MW 66.5 kDa, pI 4.7), and γ-globulin, MW 155-160 kDa, pI 6.85) were all obtained from Sigma-Aldrich (Burlington, MA, USA). N-tert-butylacrylamide (TBAm) was purchased from Tokyo Chemical Industry (Tokyo, Japan). Tetraethylorthosilicate (TEOS) was obtained from Alfa Aesar (Ward Hill, MA, USA), 25% ammonia solution from Riedel de Haen (Seelze, Germany), horseradish peroxidase isoenzyme C (HRP C; MW 44 kDa, pI 8.8) was from Roche (Mannheim, Germany), 1-ethyl-3-(3-dimethylaminopropyl) carbodiimide hydrochloride (EDC) was from Thermo Fischer Scientific (Rockford, IL, USA). Gradient grade acetonitrile, ethanol and hexane were from Merck (Darmstadt, Germany). 85% orto-phosphoric acid was obtained from VWR International (Radnor, PA, USA).

All chemicals were used as received, except the NIPAm, which was recrystallized from hexane and AAc was passed through an aluminum oxide inhibitor remover column (Sigma-Aldrich) before use. Ultrapure water was produced by a Millipore Direct-Q system (Merck).

### 2.2. Synthesis of Lys-PMNPs

#### 2.2.1. Synthesis of Magnetite NPs

Monodispersed Fe_3_O_4_ NPs were prepared through a solvothermal reaction according to the literature [50]. Briefly, FeCl_3_·6H_2_O (20.2 g) and NaOAc (54.0 g) were dissolved in ethylene glycol (600 mL) and PEG 4000 (20.2 g) and vigorously stirred for 30 min. The obtained yellow solution was transferred to a Teflon-lined stainless-steel autoclave and stirred for 24 h at 200 °C. After that the autoclave was cooled to room temperature. The obtained Fe_3_O_4_ NPs were separated from the reaction mixture using a Nd-Fe-B permanent magnet, washed several times with water and ethanol, and finally dried under vacuum overnight.

#### 2.2.2. Synthesis of Fe_3_O_4_@SiO_2_ Nanospheres

Fe_3_O_4_@SiO_2_ nanospheres were prepared by the modification of the commonly used sol-gel method [51]. Briefly, Fe_3_O_4_ nanoparticles (5.0 g) were redispersed in the mixture of PEG 400 (5 g) and ultrapure water (175 mL) by sonication for approximately 30 min. Subsequently, under continuous shaking at 700 rpm, 25% ammonia solution (30 mL) and TEOS (12 mL) were consecutively added into the reaction mixture. The reaction proceeded at room temperature for 24 h under continuous shaking. The resulting product, Fe_3_O_4_@SiO_2_, was obtained by magnetic separation and washed with ultrapure water three times then with ethanol three times and finally dried under vacuum overnight.

#### 2.2.3. Synthesis of Amine-Modified Fe_3_O_4_ NPs (Fe_3_O_4_@SiO_2_-NH_2_)

Fe_3_O_4_@SiO_2_ NPs (500 mg) were dispersed in ethanol (5 mL) and PEG 400 (100 mg) and the mixture was sonicated for 30 min. After that, 25% ammonia solution (50 µL) was added to get a homogenous solution. The resulting mixture was shaken vigorously at 500 rpm, and a mixture of APTMOS (0.52 mL) and ethanol (5 mL) was added dropwise. The suspension was shaken at 500 rpm at room temperature for 24 h The surface-grafted Fe_3_O_4_@SiO_2_–NH_2_ nanoparticles were collected by magnet and washed three times with ethanol and three times with water and the particles were dried under vacuum overnight [52].

#### 2.2.4. Coupling of the RAFT Agent

The RAFT agent, 4-cyano-4-(phenylcarbonothioylthio)pentanoic acid was immobilized through its carboxylic group to the NH_2_-modified Fe_3_O_4_@SiO_2_-NH_2_ nanoparticles by EDC to allow controlled polymer growth from the surface of the particles [53]. Fe_3_O_4_@SiO_2_–NH_2_ nanoparticles (254 mg) were mixed with RAFT (76.14 µmol) and EDC (152.24 µmol) in 2.6 mL 80: 20 v/v% acetonitrile: water and were agitated for 2 h at room temperature. The obtained Fe_3_O_4_@SiO_2_@RAFT particles were washed with acetonitrile three times and dried under vacuum overnight.

#### 2.2.5. Preparation of the Polymer Shell

Thermoresponsive polymer shell on the particles was formed by dispersing Fe_3_O_4_@SiO_2_@RAFT nanoparticles (32 mg) into 64 mL aqueous solution of NIPAm (249.5 mg, 53 mol%), AAc (14 µL, 5 mol%), TBAm (211.6 mg, 40 mol%) and BIS (12.8 mg, 2 mol%). The total monomer concentration was 65 mM. Argon gas was bubbled through the reaction mixture for 60 min. Following the addition of APS (38.4 mg) and either TEMED (24.8 µL) or NaHSO_3_ (17.3 mg), the polymerization was carried out at room temperature to obtain Lys-PMNP-TEMED and Lys-PMNP, respectively. In the end the particles were collected with a magnet and washed with water five times.

### 2.3. Characterization

Morphology and structure of the products Fe_3_O_4_, Fe_3_O_4_@SiO_2_, Fe_3_O_4_@SiO_2_-NH_2_, Fe_3_O_4_@SiO_2_-NH_2_-RAFT, Lys-PMNP and Lys-PMNP-TEMED were observed in a FEI Tecnai G^2^ 20 X-Twin transmission electron microscope (TEM) (FEI, Hillsboro, OR, USA) using 200 kV accelerating voltage and a Hitachi S-4800 scanning electron microscope (SEM) (Hitachi High-Tech Co., Ltd., Fukuoka, Japan) using 30 kV accelerating voltage. Infrared spectra were taken by a Spectrum Two Fourier-transform infrared (FTIR) spectrometer (Perkin Elmer) (Liantrisant, UK). Thermogravimetric (TG) analysis was carried out using a Q600 thermal analyzer (TA Instruments Inc., New Castle, DE, USA) in air. X-ray diffraction (XRD) patterns were recorded by an X’pert Pro MPD X-ray diffractometer using CuKα radiation (PANalytical, Almelo, The Netherlands). The zeta potential of the NPs in aqueous solution was measured with a Zetasizer Nano-ZS instrument (Malvern Instruments, Malvern, UK). To quantify the amino groups on the particles during synthesis, a BioTek instrument (Winooski, VT, USA) was used.

### 2.4. Equilibrium Binding Measurement of Lys-PMNPs in Buffer

Equilibrium binding assays were performed by mixing Lys-PMNPs with different concentrations of lysozyme in phosphate buffer (PB; 50 mM pH = 7 or 10 mM pH 8.4) and incubating the solution at 30 °C. Afterwards, the Lys- PMNPs were separated by an external magnetic field. The concentration of lysozyme in the supernatant was determined by HPLC measurement. The bound lysozyme concentration (Q) on the particles was calculated using the following formula:

$$Q=\left(C_{0}-C_{e}\right)*V/m$$

where C_0_ and C_e_ (µg/mL) are the initial concentration and the equilibrium concentration of lysozyme, respectively, V (mL) is the volume of the solution, and m (g) is the mass of the Lys-PMNPs.

### 2.5. Release of Lysozyme from the Lys-PMNPs

After lysozyme was absorbed onto the nanoparticles, the supernatant was discarded and PB with or without NaCl additive was added. The mixture was incubated for 2 h at 5 °C to release the Lys. Following magnetic separation, the concentration of lysozyme in the supernatant was determined by HPLC.

### 2.6. Measurement of Selectivity

Selectivity of the Lys-PMNPs was studied by incubating the nanoparticles (1.6 mg/mL) in HRP, avidin, BSA, albumin from chicken egg, HSA, immunoglobulin or Cyt C protein solutions (50 μg/mL in 50 mM PB pH = 7 or in 10 mM PB pH 8.4). The mixture was incubated for 1 h at room temperature, and after magnetic separation of the Lys-PMNPs, the proteins were quantitated in the supernatant by HPLC.

### 2.7. Binding and Release of Lys-PMNPs in Urine

Human urine acquired from a healthy volunteer was used to study the applicability of the Lys-PMNPs for the specific recognition of lysozyme in real samples. Without any pretreatment, human urine was spiked with lysozyme at different concentration levels. Equilibrium binding assay was performed by mixing Lys-PMNPs (2 mg) into 225 µL 10 mM PB (pH = 8.4) and adding 25 μL spiked urine, thereby achieving 10 times dilution of urine. The samples were incubated for 30 min at 30 °C with continuous shaking at 500 rpm. Afterwards, Lys-PMNPs were separated in a MagnetoPURE magnetic separator block (Chemicell, Berlin, Germany). The concentration of lysozyme in the supernatant was determined by HPLC measurement. To release Lys from the nanoparticles, the supernatant was discarded and the particles were washed two times with 250 µL water, then 250 µL PB (50 mM, pH = 7) containing 0.2 M NaCl was added. The mixture was agitated intermittently for 2 h at 5 °C to release Lys. After magnetic separation the desorbed lysozyme in the supernatant was determined by HPLC measurement. Unknown urine samples were quantitated in the same way.

### 2.8. HPLC Analysis

A Flexar FX-20 UHPLC system (Perkin Elmer, Shelton, CT, USA) with UV detector was used for the quantitation of lysozyme in the binding experiments. The chromatographic column was a Zorbax 300 SB-C8 (150 × 4.6 mm i.d., 5 μm mm, Agilent) thermostated at 37 ± 1 °C. Mobile phase solvent A was 0.1% trifluoroacetic acid in water and solvent B was acetonitrile with 0.1% TFA. The flow rate was 1.6 mL/min. The following gradient was used in the separation: from 0 to 5 min B% was increased from 30% to 45%; within 0.1 min B% was decreased to the initial 30% and held for 5 min. The injection volume was 30 µL and the detection wavelength was 280 nm. Before measurement, all samples that did not contain it already, were supplemented with 0.2 M NaCl to minimize adsorption of lysozyme onto the HPLC system parts and sample vials.

### 2.9. Measurement of Urine Samples with Micrococcus lysodeikticus Assay

Freeze-dried *Micrococcus lysodeikticus* cells were resuspended at 150 μg/mL concentration in PB (50 mM, pH 6.2). A solution containing 100 μL lysozyme was added to a 2500 μL cell suspension, and the cell lysis was followed at 25 °C by measuring the decrease in absorbance at 450 nm using a UV-Vis spectrophotometer (JASCO V-550, JASCO International Co. Ltd., Tokyo, Japan). The initial slope of absorbance decrease was used as a measure of lysozyme activity. Lysozyme calibration standards in the 2.5–20 µg/mL range were prepared in 50 mM PB, pH 7 containing 0.2 M NaCl. Unknown urine samples were diluted 10 times in 10 mM PB, pH 8.4 containing 0.2 M NaCl and further diluted, if necessary, to fall into the calibration range.

## 3. Results and Discussion

### 3.1. Preparation and Characterization of Poly(N-isopropylacrylamide-co-N-tert-butylacrylamide co-acrylic acid) Coated Magnetic Nanoparticles (Lys-PMNPs)

**Scheme 1:**
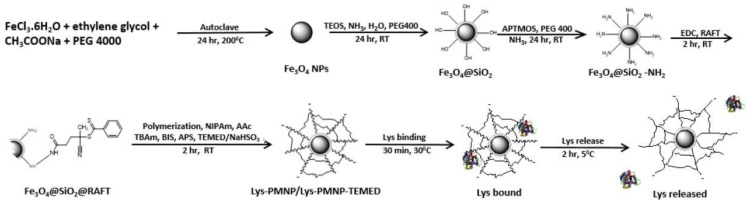
A schematic representation of Lys-PMNP preparation.

Lys-PMNPs were prepared by synthesizing a magnetic core, followed by the layer-by-layer formation of the polymer coating (Scheme 1).

Narrow-disperse magnetic nanoparticles prepared by a solvothermal method were first covered with a thin silica layer and functionalized with amine functional groups. To these, a RAFT-agent, 4-cyano-4(phenylcarbonothioylthio) pentanoic acid was attached that enabled the growth of the polymer shell from the surface of the particles in a controlled manner. A thermoresponsive poly(NIPAm) hydrogel with balanced combination of negatively charged (AAc) and hydrophobic (TBAm) monomers was grown onto the MNPs by reversible addition fragmentation chain transfer polymerization using a low percentage of BIS crosslinking monomer. The composition of the polymer layer had already been optimized for selective binding of lysozyme and the synergistic role of hydrophobic and electrostatic forces on the binding strength has been demonstrated [16]. Polymerization was carried out at room temperature using an ammonium persulfate/NaHSO_3_ redox initiator system. Our earlier studies have shown that this initiator system is superior to the commonly used APS/TEMED system because the resulting polymer has much more uniform monomer distribution. Moreover, the polymer prepared in this way shows higher affinity to lysozyme [48]. The RAFT agent immobilized onto the Fe_3_O_4_@SiO_2_-NH_2_ particles also assisted the formation of a thin polymer layer with homogeneous monomer distribution by controlling the polymerization rate. One batch of Lys-PMNP was synthesized using the APS/TEMED initiator system for comparison (Lys-PMNP-TEMED). The success of each synthesis step was confirmed by SEM, TEM, FTIR, TG, XRD and zeta potential measurement and by measuring the free amino groups by a colorimetric method.

**Figure 1:**
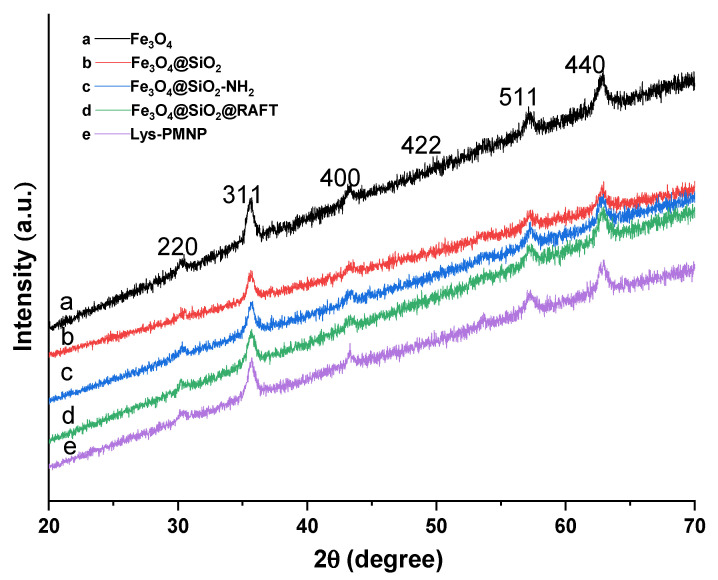
X-ray diffraction spectra of Fe_3_O_4_ (**a**), Fe_3_O_4_@SiO_2_ (**b**), Fe_3_O_4_@SiO_2_-NH_2_ (**c**), Fe_3_O_4_@SiO_2_@RAFT (**d**) and Lys-PMNP (**e**).

The crystal structure of Fe_3_O_4_, Fe_3_O_4_@SiO_2_, Fe_3_O_4_@SiO_2_-NH_2_, Fe_3_O_4_@SiO_2_@RAFT and Lys-PMNP was investigated by using XRD analysis, and the spectra are presented in Figure 1. They show the six characteristic peaks of magnetite marked by their indices (220, 311, 400, 422, 511, and 440) [41] which were obtained in the 2θ range of 20–70. It is clear that the position of the diffraction peaks was invariant, indicating that the crystalline structure of Fe_3_O_4_ was unchanged after the encapsulation into SiO_2_, and after further modifications including the polymer layer formation.

In order to demonstrate how the magnetization characteristics of the magnetite changed after the consecutive surface modifications, we have taken a practical approach. We compared, how fast the Lys-PMNPs and bare Fe_3_O_4_ NPs are collected by a magnet, with this process being shown in a video (see Video S1 in the Supplementary Materials). The bare magnetite nanoparticles could be collected much faster than Lys-PMNP due to the surface modifications on the latter. Still, Lys-PMNPs could also be collected with a reasonable speed, enabling their use as magnetically separable sorbents.

**Figure 2:**
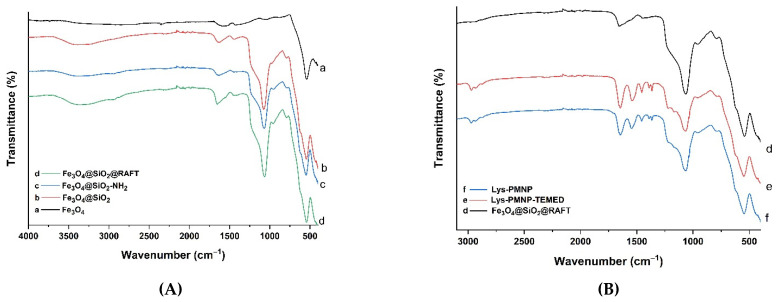
(**A**) FT-IR spectra of Fe_3_O_4_ (**a**), Fe_3_O_4_@SiO_2_ (**b**), Fe_3_O_4_@SiO_2_-NH_2_ (**c**), Fe_3_O_4_@SiO_2_@RAFT (**d**) in the 4000–400 cm^−1^ range; (**B**) FT-IR spectra of Fe_3_O_4_@SiO_2_@RAFT (**d**), Lys-PMNP-TEMED (**e**) and Lys-PMNP (**f**) in the 3100–400 cm^−1^ range.

FT-IR was used to characterize the chemical composition of Fe_3_O_4_, Fe_3_O_4_@SiO_2_, Fe_3_O_4_@SiO_2_-NH_2_, Fe_3_O_4_@SiO_2_@RAFT, Lys-PMNP and Lys-PMNP-TEMED and the spectra are presented in Figure 2A,B.

All six spectrum curves had the (Fe–O) characteristic absorption peak at 540 cm^−1^, indicating that Fe_3_O_4_ was successfully encapsulated in the polymer and the magnetite nanoparticles were not affected after the modifications. In Figure 2b–f, the strong absorption peaks at 1100 cm^−1^ and 794 cm^−1^ were attributed to symmetric and asymmetric stretching vibrations of (Si-O) respectively, and the broad absorption peaks at 1662 cm^−1^ and 3400 cm^−1^ to (–OH) silanol group vibrations, indicating that the Fe_3_O_4_ core had been successfully coated with the silica layer. The FT-IR spectrum of the Fe_3_O_4_@SiO_2_-NH_2_ and Fe_3_O_4_@SiO_2_@RAFT particles (Figure 2A(c,d), respectively) show weak absorption peaks between 2800–3100 cm^−1^ which are related to the stretching vibration mode of C–H bonds in aliphatic and/or aromatic groups. The weak band at 1658 cm^−1^ in the Fe_3_O_4_@SiO_2_@RAFT is dedicated to C=O stretching vibration in the amide group. The very weak absorption at 1444 cm^−1^ is due to the C=C bond in the aromatic ring inferring the attachment of the RAFT agent.

In the polymer coated particles (Figure 2B(e,f)) the absorption peaks at 1540 cm^−1^ are caused by (N-H) bending, at 1644 cm^−1^ by (C=O) stretching vibrations of the amide groups. The peaks at 2826 cm^−1^ and 2925 cm^−1^ are (C-H) stretching vibrations. Absorption peaks at 1392 cm^−1^ and 1365 cm^−1^ are characteristic of symmetrical stretching vibrations in carboxylic groups. These confirm the successful synthesis of the polymer shell on the Fe_3_O_4_@SiO_2_@RAFT particles.

**Figure 3:**
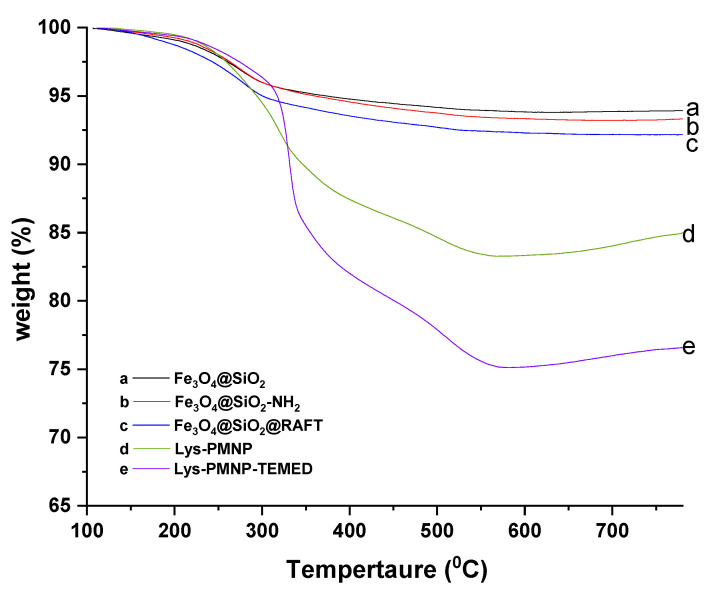
TG curves of Fe_3_O_4_@SiO_2_ (**a**), Fe_3_O_4_@SiO_2_-NH_2_ (**b**), Fe_3_O_4_@SiO_2_@RAFT (**c**), Lys-PMNP (**d**) and Lys-PMNP-TEMED (**e**).

The weight loss of Fe_3_O_4_@SiO_2_, Fe_3_O_4_@SiO_2_-NH_2_, Fe_3_O_4_@SiO_2_@RAFT, Lys-PMNP and Lys-PMNP-TEMED was studied from 100 °C to 750 °C by thermogravimetry and shown in Figure 3.

Fe_3_O_4_@SiO_2_, Fe_3_O_4_@SiO_2_-NH_2_ and Fe_3_O_4_@SiO_2_@RAFT showed a weight loss of 6.1%, 6.5% and 7.6%, respectively between the 200 °C and 550 °C range, indicating the decomposition of some residual organic contaminants and the loss of water from the surface–OH groups. Compared with Fe_3_O_4_@SiO_2_@RAFT, Lys-PMNP and Lys-PMNP-TEMED showed an additional weight loss of 9.0%, and 16.8%, respectively in the same temperature range due to the decomposition of the grafted polymer shell.

The surface charge of the nanoparticles was characterized by zeta potential measurement. The measurements in water showed zeta potentials of 31.4 ± 2.8 mV for Fe_3_O_4_, and a more negative −3.4 ± 3.3 mV for Fe_3_O_4_@SiO_2_ because of the negatively charged silanol groups. The zeta potential of the amine-functionalized Fe_3_O_4_@SiO_2_-NH_2_ particles was positive (30.7 ± 0.3 mV) because of the incorporation of protonated amino groups. The surface amino group concentration was 147 nmol/mg quantitated by the ninhydrin colorimetric assay (see Section S1 in the Supplementary Materials).

**Figure 4:**
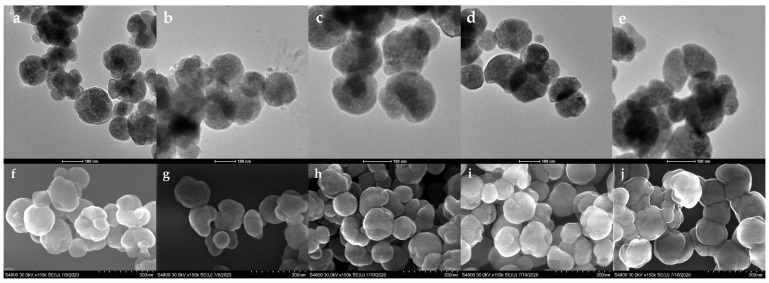
TEM and SEM images of the core–shell magnetic nanoparticles. (**a**–**e**) are TEM and (**f**–**j**) are SEM images of Fe_3_O_4_, Fe_3_O_4_@SiO_2_, Fe_3_O_4_@SiO_2_-NH_2_, Lys-PMNP and Lys-PMNP-TEMED, respectively.

The morphology of the Fe_3_O_4_, Fe_3_O_4_@SiO_2_, Fe_3_O_4_@SiO_2_-NH_2_, Lys-PMNP and Lys-PMNP-TEMED particles was characterized by TEM (Figure 4a–e) and SEM (Figure 4f–j) measurements.

The particles displayed a globular shape with a relatively narrow size distribution. The Fe_3_O_4_ core diameter was ≈130 nm, the thickness of the silica shell was 5.4 ± 0.9 nm based on the TEM measurements. Figure 4b shows the core–shell structure of magnetic Fe_3_O_4_@SiO_2_ NPs with a thin silica shell coating. The SiO_2_ layer, however, was not uniformly coated on the Fe_3_O_4_ NPs. In Figure 4c it can be observed that after the modification with 3-aminopropyltrimethoxysilane, the Fe_3_O_4_@SiO_2_-NH_2_ particles are covered with a uniform silica layer, the average thickness of which is 5.4 ± 0.9 nm. The surface modification with the polymer shell using either the APS/NaHSO_3_ or the APS/TEMED initiator system (Figure 4d,e,i,j) is not explicitly visible by TEM or SEM.

The polymer shell thickness was roughly estimated from the TG measurements (see Figure 3) to be 17 nm for Lys-PMNP and 29 nm for Lys-PNMP-TEMED (for calculation of the polymer shell thickness see Section S2 in the Supplementary Materials).

After washing, the particles formed a stable colloid in aqueous solution. Lys-PMNPs could be quickly separated with a magnet and redispersed easily, making their use very simple in sample preparation. The Lys-PMNP-TEMED particles, however, could be collected more slowly due to their thicker polymer shell (see Video S2 in the Supplementary Materials). Further studies have shown that these particles have inferior lysozyme binding affinity compared with the ones prepared with the APS/NaHSO_3_ initiator system (data not shown). In the followings, different parameters were studied which influenced the lysozyme binding property of Lys-PMNPs.

### 3.2. Effect of Time on the Binding of Lys-PMNPs

To investigate the recognition properties of the prepared Lys-PMNPs, equilibrium binding measurements were carried out by varying the experimental conditions.

**Figure 5:**
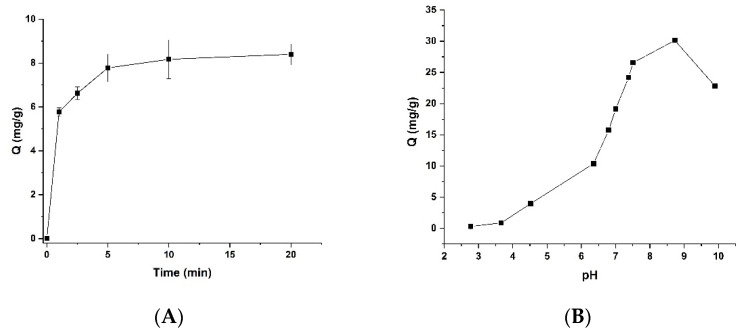
(**A**) Lysozyme binding on Lys-PMNPs after increasing incubation times. (Lys-PMNP concentration is 1.6 mg/mL; lysozyme concentration is 25 µg/mL in 50 mM PB, pH = 7; (*n* = 3)); (**B**) effect of pH on the lysozyme binding capacity of Lys-PMNPs. (1.6 mg/mL Lys-PMNP was incubated with 50 µg/mL lysozyme in different buffers. Citrate-phosphate buffers were used between pH 2.8 and 7.4 and carbonate-bicarbonate buffers at pH 8.4 and 9.7. The buffer concentration was 10 mM in each case. The pH of the buffered lysozyme solution was measured and plotted on the *x*-axis. (*n* = 3)).

Lys-PMNPs were immersed into 25 µg/mL lysozyme solution (50 mM PB, pH = 7) and the lysozyme binding was measured after different time intervals. The bound concentration with incubation time is plotted in Figure 5A.

It can be seen that the protein binds to the nanoparticles extremely fast, with maximum binding achieved in only 5 min. Other lysozyme-selective polymer-based MNPs described in the literature generally show much slower binding kinetics, with equilibration times from 40 to 180 min being typical [18,37,38,39,40,41,43,44]. We presume that the observed fast binding kinetics is due to the strong attractive Coulomb forces between the negatively charged surface of Lys-PMNPs and the positively charged protein. As opposed to this, in MIP coated MNPs, the diffusion of the protein to the imprinted sites is probably a limiting step in the binding process.

### 3.3. Effect of Buffer Ionic Strength and pH on the Protein Binding of Lys-PMNPs

Due to the partly electrostatic nature of lysozyme binding, we have investigated the role of the buffer concentration in the binding process. The protein binding was measured in 25 µg/mL lysozyme dissolved in 10 or 50 mM PB (pH = 7). The nanoparticles bound only 52% of the lysozyme in 50 mM PB (ionic strength: 0.3 M). In contrast, when the buffer concentration was decreased to 10 mM (ionic strength: 0.06 M), all the lysozyme was bound to the nanoparticles. This indicated that the buffer concentration/ionic strength plays a significant role in the binding process. It is acting against the electrostatic attractive forces in the lysozyme-nanoparticle interaction through shielding of the surface charges.

The effect of pH on the lysozyme binding property of the Lys-PMNPs was investigated in 50 µg/mL lysozyme in buffered solutions. The buffer concentration was kept low, so that the ionic strength would not interfere with the protein binding. The bound lysozyme concentration as a function of the pH is shown in Figure 5B.

The bound concentration on the Lys-PMNPs increases to a large extent when going from low pH to neutral and reaches a maximum between pH 8–9. Further increase in the pH leads to decreased protein binding. This can be explained with the protonation degree of lysozyme and the acrylic acid groups in the polymer. The pK_a_ of acrylic acid in Lys-PMNPs is estimated to be 5.8 based on the measurements of Ito et al. [54], while the pI value of lysozyme is 11.35 [55]. Below pH ≈ 3.8 the acrylic acid moieties are fully protonated, therefore they cannot provide any interaction point for the lysozyme and the binding is indeed very low. Increasing the pH, the acrylic acid groups start to deprotonate and provide more and more negative charges to bind the positively charged lysozyme. Above pH ≈ 7.8 they are fully deprotonated and offer maximum binding capacity. However, as the pH is approaching the isoelectric point of lysozyme, there are less and less positively charged groups on the protein surface, therefore the binding capacity decreases.

### 3.4. Adsorption Isotherms

**Figure 6:**
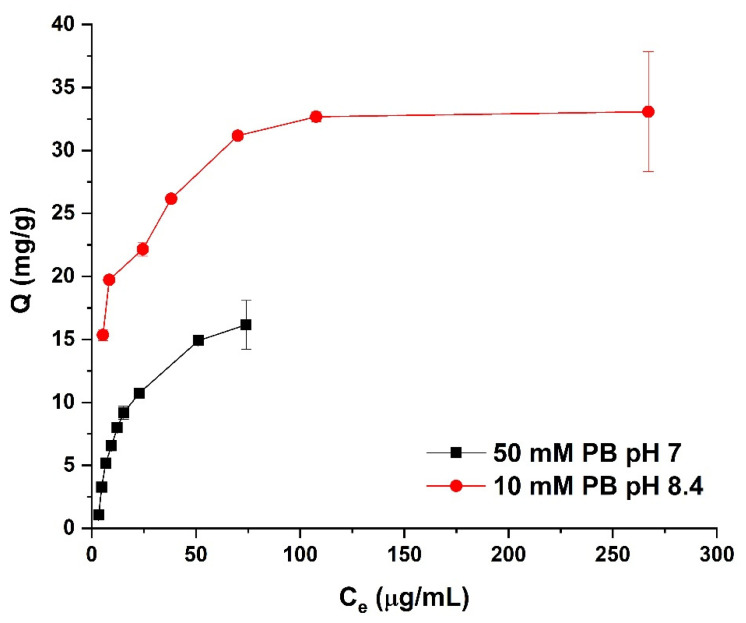
Adsorption isotherms of Lys-PMNPs in buffered solution of lysozyme. (1.6 mg/mL Lys-PMNP was incubated with 5, 15, 30, 40, 60, 80, 120, 160 and 320 µg/mL lysozyme in 10 mM PB pH 8.4, and in 5, 10, 15, 20, 25, 30, 40, 75 and 100 µg/mL lysozyme in 50 mM PB, pH 7 (*n* = 3)).

Lys-PMNPs were incubated with different concentrations of lysozyme either in 10 mM PB, pH 8.4 or in 50 mM PB, pH 7. After equilibration, the concentration of the unbound protein in the supernatant was measured by HPLC and the concentration of the bound lysozyme was calculated. The two adsorption isotherms are shown in Figure 6.

Both isotherms showed saturation at high concentrations of lysozyme, and in 10 mM PB (pH 8.4), as could be expected, the binding capacity was much higher than in 50 mM PB (pH 7). In 10 mM PB, below 30 µg/mL initial concentration, the equilibrium concentration in the supernatant was below the detection limit. We have roughly estimated the maximum binding capacity in the two buffer systems by fitting a Langmuir isotherm on the measurement points. In 50 mM PB (pH 7) the lysozyme binding capacity was 21.4 ± 1.3 mg/g, while in 10 mM PB (pH 8.4) it amounted to 33.8 ± 1.4 mg/g. From the latter result the average surface area covered per lysozyme molecule was roughly calculated using the diameter and shell thickness of the Lys-PMNPs obtained from the SEM, TEM and TG measurements, the corresponding densities (ρ_Fe_3___O_4__: 5.2 g/cm^3^; ρ_SiO_2__: 2.2 g/cm^3^; ρ_polymer_: 0.5 g/cm^3^ [56]) and the molecular weight and size of lysozyme. At the maximum binding capacity in 10 mM PB (pH 7) the surface area taken up by one lysozyme molecule was approximately 9.2 nm^2^. Lysozyme has an elongated shape and can adsorb to surfaces along its short or long axis having a footprint of 4.9 or 9.7 nm^2^, respectively [57]. The latter value shows a close agreement with our results, suggesting that in our system lysozyme is strongly adsorbed on the surface along its long axis and completely covers the surface as a monolayer.

### 3.5. Selectivity towards Different Proteins

**Figure 7:**
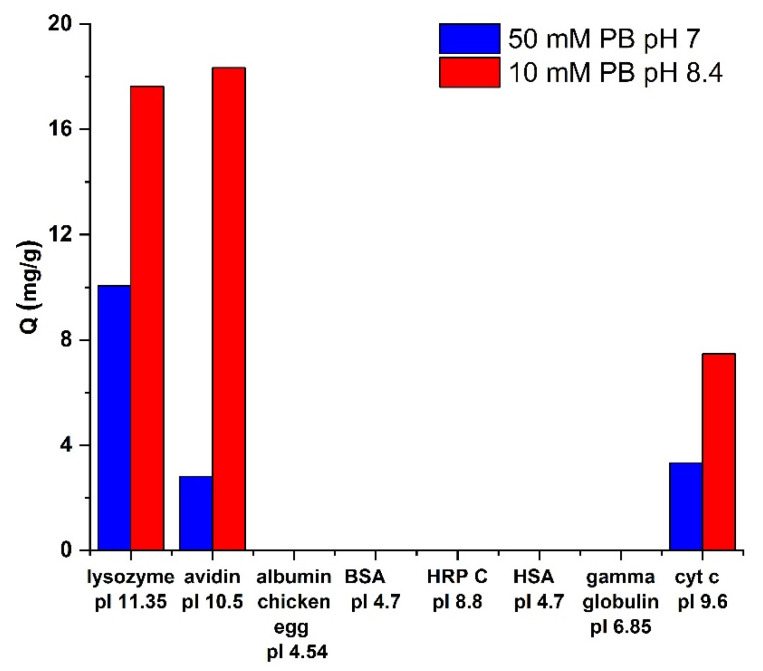
Selectivity of Lys-PMNPs towards other proteins at room temperature in different buffer systems. (1.6 mg/mL Lys-PMNP was incubated with 50 µg/mL protein solution in 50 mM PB, pH 7 or in 10 mM PB, pH 8.4).

Selectivity of the Lys-PMNPs towards various proteins was assessed by comparing the amount of bound protein in 50 µg/mL buffered solutions (50 mM, pH 7 or 10 mM pH 8.4) of lysozyme, avidin, HRP C, BSA, albumin from chicken egg, HSA, γ-globulin and cyt C at room temperature. The results are shown in Figure 7.

It can be seen that the Lys-PMNPs show high selectivity towards BSA, albumin from chicken egg, HSA and γ-globulin having acidic or neutral, and HRP C with weakly basic isoelectric point. These proteins do not bind to the particles at all under the given circumstances. Cyt C and avidin with more basic pI values show moderate binding to the nanoparticles compared with lysozyme in the high ionic strength buffer. In low ionic strength buffer, however, the selectivity against avidin drastically decreases. This might be explained by the much bigger size of avidin offering more electrostatic interaction points (the density of positively charged amino groups on the two proteins is very similar) and consequently stronger binding in low ionic strength media. This indicates that the selectivity of the nanoparticles towards positively charged proteins is also substantially influenced by the ionic strength.

We have also investigated the binding of lysozyme in the presence of clinically relevant proteins (HSA and γ-globulin) that might exhibit high urine concentrations in different diseases accompanied by proteinuria. The concentration of the potentially interfering proteins was chosen as the upper limit in their abnormal range. Lys-PMNPs were incubated in a mixed protein solution containing 50 µg/mL lysozyme, 200 µg/mL HSA and 500 µg/mL γ-globulin in 10 mM PB, pH 8.4. It was found that even in the presence of high concentrations of interfering proteins, the nanoparticles bound exactly the same amount of lysozyme as without them (17.63 mg/mg vs. 17.50 mg/g). This indicates that the nanoparticles show extremely high selectivity, which potentiates their use in clinical analysis of lysozyme in the presence of other highly abundant proteins.

### 3.6. Thermally Modulated Binding and Release of Lysozyme

**Figure 8:**
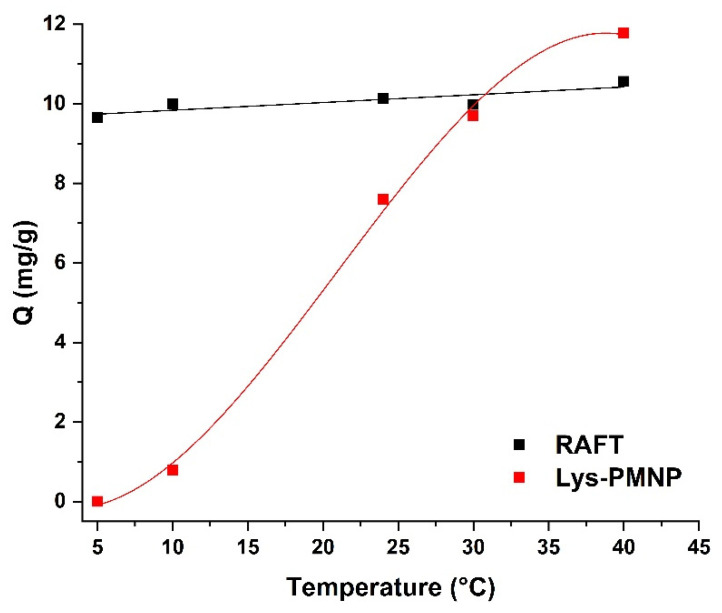
Effect of temperature on the lysozyme binding capacity of Lys-PMNPs and Fe_3_O_4_@SiO_2_@RAFT particles. (1.6 mg/mL Lys-PMNP or Fe_3_O_4_@SiO_2_@RAFT particles were incubated with 50 µg/mL lysozyme in 50 mM PB, pH 7).

The effect of temperature on the lysozyme binding properties of the nanoparticles was investigated at four different temperatures. As a comparison, the Fe_3_O_4_@SiO_2_@RAFT particles were also included in this set of experiments. The lysozyme binding capacity of the nanoparticles is shown in Figure 8.

The protein binding of Lys-PMNPs shows a strong temperature dependence as could be expected from the thermoresponsivity of the poly(N-isopropylacrylamide-co-N-tert-butylacrylamide-co-acrylic acid) shell. This polymer is in a highly swollen state showing hydrophilic properties below its volume phase transition temperature (approx. 20 °C [58]) and in a more hydrophobic, collapsed state above it. This is reflected in its affinity to lysozyme, i.e., no protein is bound at 5 °C, while increasing the temperature up to 40 °C results in more and more lysozyme becoming bound. This property of the Lys-PMNPs can be exploited in a sample cleanup process.

In Figure 8, it can be observed that the Fe_3_O_4_@SiO_2_@RAFT particles without the polymer shell also show high protein binding (similarly to the Lys-PMNPs at higher temperatures), but it is evident that their binding capacity is independent of temperature. This is an indirect proof that the polymer was successfully formed on the RAFT-modified nanoparticles.

**Figure 9:**
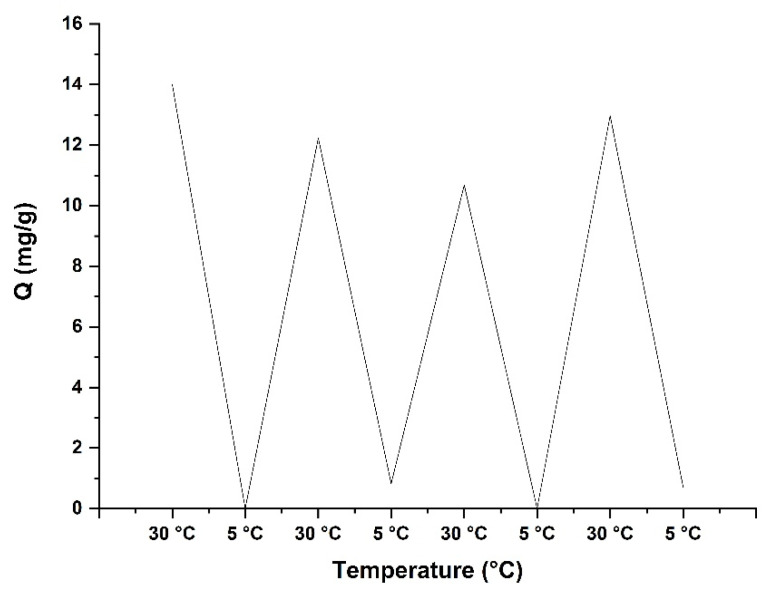
Reversibility of the temperature-induced protein binding and release. (1.6 mg/mL Lys-PMNP was incubated with 50 µg/mL lysozyme in 50 mM PB, pH 7).

We have investigated the reversibility of the protein binding and release by applying multiple temperature switches between 5 and 30 °C and quantitating the amount of free lysozyme after each temperature switch. The bound protein was plotted in Figure 9 after each cycle. The lysozyme binding and release is fully reversible even after four heating and cooling cycles.

### 3.7. Development of a Sample Cleanup Procedure for the Measurement of Lysozyme in Clinical Urine Samples

A sample cleanup procedure from urine was designed using the selective Lys-PMNPs as magnetically separable dispersive solid phase extraction medium. After lysozyme binding, the nanoparticles were washed, then the bound protein was released by thermal modulation into a buffer solution. The released lysozyme was quantified by HPLC-UV. During method development, we have targeted the 0–2000 µg/mL urine concentration range relevant in the diagnosis of monocytic and myelomonocytic leukemia [21].

First, we investigated the binding of lysozyme to the Lys-PMNPs from urine at 30 °C. Although the binding of lysozyme was proven to be very fast, as described earlier, we chose a 30 min incubation time to allow for thermal equilibration. Urine of a healthy volunteer with no measurable lysozyme was spiked to 25 µg/mL concentration and incubated with Lys-PMNPs (c = 5.3 mg/mL). Compared with binding in 50 mM PB, pH 7 where 90.7% of the added lysozyme was bound from the supernatant, in urine only 53.0% was bound. This was attributed to the high salt concentration of urine. Desalting of urine by dialysis, centrifugal ultrafiltration or size exclusion column led to partial loss of lysozyme, therefore, for further measurements urine was diluted ten times to decrease its ionic strength and facilitate lysozyme binding. From the ten-times diluted urine matrix 94.1% of the added lysozyme was bound under the same circumstances.

The concentration of Lys-PMNPs (i.e., the phase ratio) in the binding step was crucial concerning the recovery of the sample pretreatment process. High phase ratios facilitated the binding of lysozyme. Among 1.6, 5.3 and 8 mg/mL phase ratios, 8 mg/mL resulted in almost 100% (96.2 ± 3.15%) binding for 1000 µg/mL spiked urine samples (100 µg/mL diluted urine samples) and in 73.8 ± 4.6% binding for the 2000 µg/mL spiked urine samples (200 µg/mL diluted urine samples).

After the preconcentration from urine, the particles have to be washed to remove any residual matrix components, therefore 50 mM PB, pH 7 and pure water were tried as washing solvents. The volume of the washing solvent was the same as that of the urine sample. After the binding step, the supernatant was removed and the washing solvent was briefly vortexed with the particles. Fifty mM PB removed approximately 38% of the bound protein within less than 1 min contact time, while water did not remove any lysozyme. However, after one wash with water, the wash solution had considerable UV absorbance due to other urine matrix components. Therefore, the particles were washed two times with water to fully remove these contaminants.

Finally, we optimized the release of lysozyme. First, 50 µg/mL lysozyme in 10 mM PB, pH 8.4 was incubated with 1.6 mg/mL Lys-PMNPs for 30 min at 30 °C then the particles were washed with water. In the release step a buffer was pipetted over the Lys-PMNPs and a 1 h incubation at 4 °C followed, which enabled the thermoresponsive polymer shell to swell and release the bound lysozyme. To promote protein release, different buffers, additives, and phase ratios were tried. The recovery of lysozyme in the release step (i.e., the percentage released relative to the bound protein) is listed in Table 1 for different release solutions and different phase ratios.

As could be expected from the binding isotherms, protein release was more efficient in 50 mM PB pH 7, than in 10 mM PB, pH 8.4 due to the lower affinity of the Lys-PMNPs in this medium. The addition of high concentration of NaCl promoted the release of the lysozyme in each case by disrupting ionic interactions. Application of higher phase ratios in the release step compared with the binding step could have been advantageous for the preconcentration of lysozyme, something that is beneficial at low analyte concentrations. However, applying the same phase ratio in both steps increased the recovery rate, therefore, in the optimized method we have chosen a similar phase ratio as in the binding step (8 mg/mL) and 50 mM PB, pH 7 with 0.2 M NaCl. It has to be noted though, that by choosing different phase ratios in the binding and release step, the linear concentration range of the measurement can be tuned easily.

We tried to regenerate the exhausted Lys-PMNPs with different washing solvents applying low temperature (4 °C). We washed the Lys-bound PMNPs with water, 50 mM PB, pH 7 or 10 mM PB pH 8.4 with or without 0.2 M NaCl, 0.1 M acetic acid and 0.1 M HCl. All these regeneration steps were followed by thorough washing with water to remove the regeneration solution. The effectiveness of the regeneration step was assessed by rebinding lysozyme. It was puzzling to see that the regenerated particles could not rebind the same amount of protein as the unused ones, though earlier, the repeated temperature-induced binding and release cycles were fully reversible (see Figure 9). We speculate that the use of NaCl in the release step of the sample pretreatment somehow deteriorates the binding properties of the polymer shell.

The batch-to-batch reproducibility of the particles was investigated with two different Lys-PMNP preparations, both synthesized from the same batch of Fe_3_O_4_@SiO_2_ NPs. In 25 µg/mL lysozyme solution (50 mM PB, pH 7) at 1.6 mg/mL particle concentration the percentage of bound protein was 54.1 ± 5% in one case and 54.6 ± 4.3% using the other batch. This shows a surprisingly good reproducibility of the Lys-PMNP production.

### 3.8. Method Validation

The newly developed method has been partially validated following bioanalytical method validation guidelines [49].

**Figure 10:**
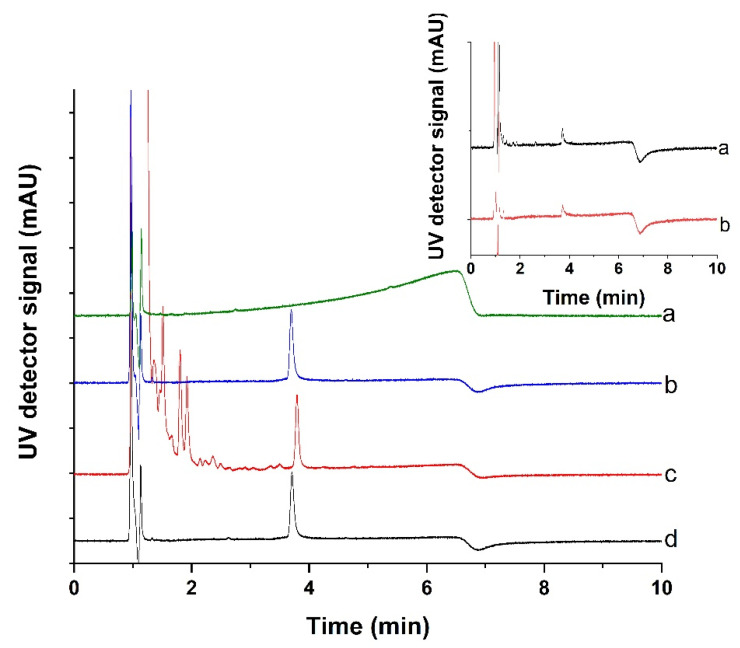
Chromatograms of a ten-times diluted blank urine sample after pretreatment (**a**), a 10 µg/mL lysozyme standard (**b**), a 10 µg/mL diluted urine sample without sample pretreatment (**c**) and a pretreated 10 µg/mL diluted urine sample (**d**). (The retention time of lysozyme is 3.7 min). The inset shows the chromatogram of a 2.5 µg/mL diluted urine sample after pretreatment (LLOQ sample) (**a**) and that of a 2.5 µg/mL lysozyme standard (**b**).

Selectivity was assessed by injecting blank and spiked diluted urine samples with and without the sample cleanup procedure, as well as lysozyme standard solutions in 50 mM PB, pH 7, with 0.2 M NaCl. The chromatograms of a blank diluted urine sample, a 100 µg/mL urine sample (10 µg/mL after ten times dilution) before and after the sample cleanup with Lys-PMNPs, and a 10 µg/mL lysozyme standard solution can be seen in Figure 10.

The method is selective, since there is no interfering peak at the retention time of lysozyme in the blank urine sample after the sample’s pretreatment (Figure 10a). By comparing the chromatograms of the spiked urine sample before and after the sample cleanup process (Figure 10c,d), the high efficiency and selectivity of the Lys-PMNPs becomes evident. In the chromatogram of the pretreated urine sample the baseline is free from any peaks as opposed to the non-treated sample where many highly absorbing substances appear besides lysozyme. The lower limit of quantification (LLOQ) is 25 µg/mL. In the inset of Figure 10, chromatograms of the LLOQ sample (25 µg/mL urine sample, 2.5 µg/mL after dilution) and a 2.5 µg/mL lysozyme standard are shown.

Linearity of the calibration was studied using eight standard solutions (50 mM PB, pH 7, 0.2 M NaCl) in the 2.5–200 µg/mL concentration range (corresponding to 25–2000 µg/mL urine concentration). Concentrations lower than 2.5 µg/mL could not be detected with HPLC-UV. We encountered a significant adsorption of lysozyme to the HPLC system parts and glass or plastic containers in the low concentration range. This adsorption was even more pronounced if NaCl was omitted from the buffer and the addition of Tween 20 surfactant could not eliminate it either. Three calibrations were measured on different days and because of the adsorption of lysozyme, two lines were fitted on each curve using the least squares method, one for the low concentration range and one for the high concentration range. The calibration equation of the lower part was y = 1.71 ± 0.16 x − 2.30 ± 0.5 (R^2^ = 0.9982 ± 0.0016) and that of the high concentration part was y = 2.18 ± 0.11 x − 13.0 ± 10.0 (R^2^ = 0.9991 ± 0.0003). Further on, these calibrations were used to quantify spiked and unknown urine samples in the 25 to 2000 µg/mL concentration range.

Intra-day repeatability was assessed by preparing five replicates of 25, 100, 250 and 1000 µg/mL spiked urine samples, which were diluted ten times and pretreated with Lys-PMNPs. After HPLC measurement their concentration was determined from the daily calibration. Intra-day precision values were established as the relative standard deviation of the results, while the accuracy was determined as the percentage measured concentration relative to the nominal concentration. The results are shown in Table 2.

Intra-day precision values ranged from 2.24 to 8.20%, while the accuracies were between 89.9 and 117.6%. These values fall in the acceptable 15% (or 20% at the LLOQ level) repeatability and 85–115% (or 80–120% at the LLOQ level) accuracy range established for bioanalytical methods.

Inter-day reproducibility was established by measuring 25; 100; 250; 1000 and 2000 µg/mL spiked urine samples on three different days using the daily calibrations. Table 3 lists the results obtained for these samples.

Inter-day precision values were between 1.08 and 5.04%, while accuracies were in the 88.8 to 111.0% range for urine samples within the 25 to 1000 µg/mL range. These values also fulfill the acceptance criteria established for bioanalytical methods.

Recovery of the sample preparation process was evaluated by comparing the HPLC peak area of spiked urine samples to that of lysozyme standard solutions having the same nominal concentration. Recovery was assessed on three different days at 100; 250; 1000 and 2000 µg/mL urine concentration levels (corresponding to 10; 25; 100 and 200 µg/mL standard solution concentration). The obtained results are shown in Table 4.

It can be seen that up to 1000 µg/mL urine lysozyme concentration, the recovery values are high, close to 100%, which also means that solution calibration can be used to quantitate the urine samples and there is no need for matrix matched calibration. The mean recovery of the bioanalytical method in this concentration range is 94.1 ± 8.1%. However, at 2000 µg/mL urine concentration, the recovery drops to 62%. This is inherent to the present optimized sample pretreatment procedure, the Lys-PMNP concentration is too low to fully bind the lysozyme. This problem could be overcome either by applying even higher nanoparticle concentration, which we did not pursue, or by diluting the urine sample into the concentration range, where the recovery is around 100%.

### 3.9. Comparison to a Reference Method by the Measurement of Unknown Samples

**Figure 11:**
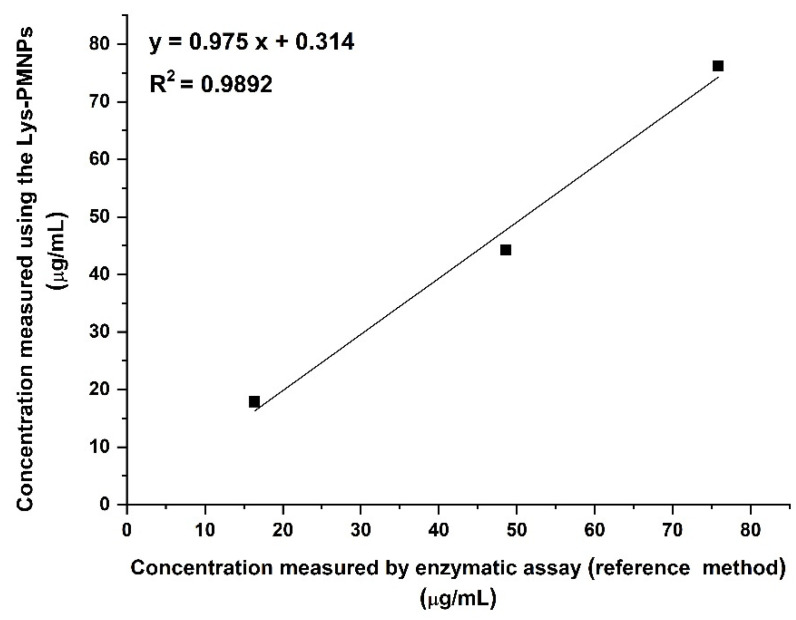
Concentrations of unknown urine samples measured using the Lys-PMNPs and by an enzymatic assay using *Micrococcus lysodeikticus* cells.

We intended to prove the clinical applicability of our method by analyzing urine samples of patients suffering from monocytic or myelomonocytic leukemia, but unfortunately due to the pandemic situation we were unable to obtain such samples from hospitals. Therefore, spiked urine samples were prepared and analyzed as unknowns both with the newly developed method and an enzymatic assay based on *Micrococcus lysodeikticus* cells. The latter reference method is a widely accepted sensitive assay for lysozyme in clinical laboratories. Results of the unknown samples obtained by the two methods were plotted against each other (see Figure 11) and a line was fitted onto them using the least squares method.

The close to 1 slope and close to 0 intercept of the regression line shows that there is no proportional error or constant bias in the new method compared to the well-established enzymatic assay. The close to 1 correlation coefficient (R^2^ = 0.9891) indicates that the results obtained by the two methods highly correlate.

The validation results and the comparison to an established clinical method shows that the selective Lys-PMNPs perform successfully as novel sample extraction medium and the optimized bioanalytical method can be used for the measurement of lysozyme in human urine samples for the clinical diagnosis of monocytic or myelomonocytic leukemia.

## 4. Conclusions

Novel thermoresponsive polymer coated magnetic nanoparticles have been synthesized and their applicability as selective extraction sorbent for proteins was demonstrated. The optimized monomer composition and initiator system endowed the Lys-PMNPs with high binding affinity and outstanding selectivity. High concentrations of the two clinically relevant proteins, human serum albumin and γ-globulin did not affect the adsorption of lysozyme onto the nanoparticles at all, which could bind and release the protein in a thermocontrolled manner. A sample pretreatment procedure has been elaborated for the measurement of lysozyme in human urine samples. The developed method has been successfully validated and compared favorably with an established clinical method. The validation results prove that the Lys-PMNP sorbent has high potential in the analysis of urine samples of monocytic and myelomonocytic leukemia patients.

## Figures and Tables

**Table 1:** Efficiency of the protein removal from Lys-PMNPs using different buffer solutions and phase ratios.

Binding Step			Release Step				
PB Concentration [mM]	pH	Phase Ratio [mg/mL]	PB Concentration [mM]	pH	Additive	Phase Ratio [mg/mL]	Recovery in the Release Step [%]
10	8.6	1.6	10	8.4	-	10.67	31.2
			50	7	-	10.67	70.0
			10	8.4	0.2 M NaCl	10.67	81.2
			50	7	0.2 M NaCl	10.67	90.6
			50	7	-	1.6	104.2

**Table 2:** Intra-day precision and accuracy of lysozyme measurement in human urine using the Lys-PMNPs (*n* = 5).

Urine Concentration [µg/mL]	Nominal Sample Concentration [µg/mL]	Measured Concentration [µg/mL]	Precision [%]	Accuracy [%]
25	2.5	2.94	3.44	117.6
100	10	9.35	8.20	93.5
250	25	25.8	4.25	103.0
1000	100	89.9	2.24	89.9

**Table 3:** Inter-day precision and accuracy of lysozyme measurement in human urine using the Lys-PMNPs (*n* = 3).

Urine Concentration [µg/mL]	Nominal Sample Concentration [µg/mL]	Measured Concentration [µg/mL]	Precision [%]	Accuracy [%]
25	2.5	2.774	1.08	111.0
100	10	9.442	3.21	94.4
250	25	24.81	5.04	99.2
1000	100	88.80	4.40	88.8
2000	200	128.8	2.25	64.4

**Table 4:** Mean recovery values at four different concentration levels (*n* = 3).

Urine Concentration [µg/mL]	Standard Solution Concentration [µg/mL]	Recovery [%]	RSD [%]
100	10	91.7 ± 3.8	4.19
250	25	103.1 ± 3.9	3.80
1000	100	87.5 ± 9.4	10.81
2000	200	62.3 ± 6.3	10.14

## Supplementary Materials

The following are available online at https://www.mdpi.com/article/10.3390/nano11113015/s1, Figure S1: Calibration curve for APTMS using ninhydrin, Table S1: Comparison of lysozyme-selective nanoparticles, Video S1: Fe_3_O_4_-LysPMNP.mp4, Video S2: Lys-PMNP-Lys-PMNP-TEMED.mp4.
