# Supplementary material for: Temperature-Responsive Magnetic Nanoparticles for Bioanalysis of Lysozyme in Urine Samples
Source: Nanomaterials (Basel). 2021 Nov 10;11(11):3015. doi: 10.3390/nano11113015 (PMC8618479; doi:10.3390/nano11113015)
Supplement: Supplementary file 1 [file nanomaterials-11-03015-s001.zip › nanomaterials-1413369-supplementary.pdf]

Supplementary Materials

Temperature-Responsive Magnetic Nanoparticles for Bioanalysis of Lysozyme in Urine Samples

Marwa A. Ahmed [1,2], Júlia Erdőssy [1] and Viola Horváth [1,3]

[1] Department of Inorganic and Analytical Chemistry, Faculty of Chemical Technology and Biotechnology,
Budapest University of Technology and Economics, H-1111 Budapest, Hungary; marwaaa@a-edu.suez.edu.eg (M.A.A.);.julia.erdossy@gmail.com (J.E.)

[2] Department of Chemistry, Faculty of Science, Arish University, 45511 El-Arish, North Sinai, Dahyet El Salam, Egypt

[3] MTA-BME Computation Driven Chemistry Research Group, H-1111 Budapest, Hungary

* Correspondence: horvath.viola@vbk.bme.hu

Section S1. Quantitation of the surface NH_2-groups

Quantitation of primary amino groups on the surface of Fe_3O_4@SiO_2-NH_2 particles was carried out in order to prove the successful layer formation from APTMOS and the consecutive modification by the RAFT agent. This was carried out by the ninhydrin colorimetric assay. The reagent mixture consisted of ninhydrin solution (140 mM in ethanol + 0.2 v/v% acetic acid) and acetate buffer (100 mM, pH 5.5) in 2:1 ratio. For calibration 400 µl ninhydrin reagent was reacted with 200 µl of various known concentrations of APTMOS (0.25; 0.4; 0.58; 0.7; 0.85; 1 and 1.15 mM in acetate buffer (100 mM, pH 5.5)) to form colored products. The samples were heated to 100 °C for 15 min. After cooling to room temperature the absorbance of the solutions was measured at 570 nm [1]. The calibration curve is shown in Figure S1.

For measuring the primary amino groups on the surface of the nanoparticles we added 600 µl reaction mixture to 3 mg of nanoparticles. The samples were treated the same way as above. After separation with a magnet the absorbance of the supernatants was measured at 570 nm. The concentration of amino groups was calculated to be 147 nmol/mg.

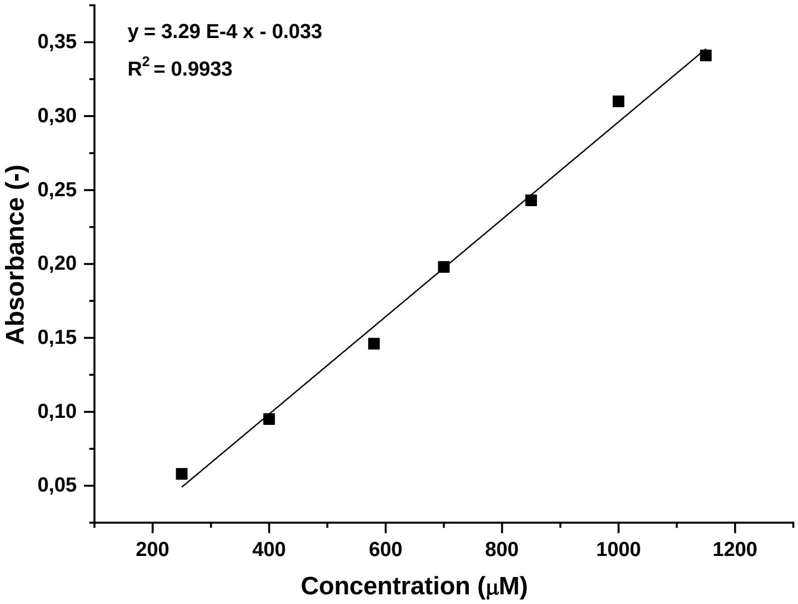

Figure S1: Calibration curve for APTMS using ninhydrin

Section S2. Calculation of the polymer shell thickness

The percentage weight loss due to the polymer shell decomposition was calculated from the TG curves for Lys-PMNPs and for Lys-PMNP-TEMED particles. From the mean diameter of the Fe_3O_4 core, the thickness of the silica shell, the density of Fe_3O_4, SiO_2 and the polymer, the thickness of the polymer shell could be calculated according to the following equation:

$$p = \frac{\sqrt[3]{\frac{\%}{100-\%} \cdot \frac{d^3 \cdot \rho_{Fe_3O_4} + ((d+2s)^3 - d^3) \cdot \rho_{SiO_2}}{\rho_{polymer}} + (d+2s)^3} - (d+2s)}{2}$$

where

d is the mean diameter of the Fe_3O_4 nanoparticles

s is the thickness of the SiO_2 shell

p is the thickness of the polymer shell

$\rho_{Fe_3O_4}$ is the density of Fe_3O_4 (5.2 g/cm^3)

ρ_{SiO_2} is the density of SiO_2 (2.2 g/cm^3)

$\rho_{polymer}$ is the density of the polymer shell (0.5 g/cm^3 in the collapsed state [2])

% is the percentage weight loss of Lys-PMNP (9.0%) or Lys-PMNP-TEMED (16.8%) due to the decomposition of the polymer shell.

Table S1: Comparison of lysozyme-selective nanoparticles

No.	Particle core	Polymer	Initiator	Selectivity achieved by	Selectivity against albumin	Equilibration time (min)	Q (mg/g)	Thermocontrolled binding and release	Biological sample	Applicability test	Reference
1	Fe_3O_4/silica	styrene-sulfonate N-isopropyl-acrylamide	azobisiso-butyronitrile at 60°C	special functional monomers	≈7	45	91	yes	human serum diluted 10 times	depletion of human serum measured by HPLC, recovery from spiked serum	[3]
2	Fe_3O_4/silica	BIS acrylamide methacrylic acid	APS, TEMED 25°C	molecular imprinting	≈5.7	100	110	no	human serum diluted 200 times	recovery from spiked human serum, measured by chemiluminescence measurement	[4]
3	Fe_3O_4/silica	BIS acrylamide methacrylic acid	APS, TEMED 25°C	molecular imprinting	≈5.7	5	110	no	human urine diluted 200 times	method validation	[5]
4	Fe_3O_4/silica	1-(α-allylacetate)-3-N-(3-aminopropyl)-imidazolium chloride BIS	APS, TEMED 25°C	molecular imprinting	≈11	150	217	no	chicken egg white	depletion of chicken egg white measured by SDS PAGE	[6]
5	Fe_3O_4/ polyacrylic acid/ chitosan-COOH	BIS acrylamide methacrylic acid (dimethylamino)ethyl methacrylate	APS, TEMED 25°C	molecular imprinting	no interference at the same concentration level	180	120	no	no	no	[7]
6	silica	BIS acrylamide methacrylic acid (dimethylamino)ethyl methacrylate	APS, TEMED 25°C	molecular imprinting	no interference at the same concentration level	5	10	no	no	no	[8]
7	chitosan wrapped carboxylated MWCNT	dopamine	-	molecular imprinting	≈21	40	400	no	egg white	depletion of chicken egg white measured by SDS PAGE	[9]

8	Fe_3O_4/ polymethyl methacrylate	dihydroxy-phenylacetic acid	-	molecular imprinting	≈13	120	125	no	no	no	[10]
9	Fe_3O_4/silica	acrylamide methacrylic acid tetraethoxysilane	azobisiso-butyronitrile	molecular imprinting	≈6	60	100	no	human urine	recovery from spiked urine	[11]
10	Fe_3O_4	dopamine	self-polymerization	molecular imprinting	≈6	720	220	no	no	no	[12]
11	Fe_3O_4/silica	2-(2-methoxyethoxy) ethyl methacrylate BIS methacrylic acid	APS, TEMED 25°C	molecular imprinting	≈28	120	204	yes	reconstitued dry human urine diluted approx. 40-90 times	depletion of human urine measured by HPLC, recovery from spiked urine	[13]
12	silica	BIS methacrylic acid	APS, TEMED 25°C	molecular imprinting	≈6.5	200	25	no	human serum diluted 50 times	depletion of human serum, measured by SDS PAGE	[14]
13	Fe_3O_4/silica	BIS NIPAm TBAm Aac	APS, SBS/TEMED 25°C	special functional monomers	no interference at four times excess of human serum albumin	5	34	yes	human urine diluted 10 times	method validation	this work

Video S1: Title video-1-Fe3O4-LysPMNP.mp4

Video S2: Title video-2-Lys-PMNP-Lys-PMNP-TEMED.mp4

References

[1] Nagy, F.; Tasnádi, G.; Balogh-Weiser, D.; Bell, E.; Hall, M.; Faber, K.; Poppe, L. Smart Nanoparticles for Selective Immobilization of Acid Phosphatases. ChemCatChem 2018, 10, 3490–3499, https://doi.org/10.1002/cctc.201800405

[2] Tokuhiro, T. Temperature Dependence of Density of Polymer Gels 1. A Pycnometry Method Applied to Poly(N-isopropylacrylamide)−Water System. J. Phys. Chem. B 1999, 103, 7097–7101, https://doi.org/10.1021/jp9843510.

[3] Zhang, M.; Qiao, J.; Qi, L. Dual-functional polymer-modified magnetic nanoparticles for isolation of lysozyme. Anal. Chim. Acta 2018, 1035, 70–76, https://doi.org/10.1016/j.aca.2018.07.019.

[4] Jing, T.; Du, H.; Dai, Q.; Xia, H.; Niu, J.; Hao, Q.; Mei, S.; Zhou, Y. Magnetic molecularly imprinted nanoparticles for recognition of lysozyme. Biosens. Bioelectron. 2010, 26, 301–306, https://doi.org/10.1016/j.bios.2010.08.044.

[5] Jing, T.; Xia, H.; Guan, Q.; Lu, W.; Dai, Q.; Niu, J.; Lim, J.-M.; Hao, Q.; Lee, Y.-I.; Zhou, Y.; et al. Rapid and selective determination of urinary lysozyme based on magnetic molecularly imprinted polymers extraction followed by chemiluminescence detection. Anal. Chim. Acta 2011, 692, 73–79, https://doi.org/10.1016/j.aca.2011.02.057.

[6] Xu, W.; Dai, Q.; Wang, Y.; Hu, X.; Xu, P.; Ni, R.; Meng, J. Creating magnetic ionic liquid-molecularly imprinted polymers for selective extraction of lysozyme. RSC Adv. 2018, 8, 21850–21856, https://doi.org/10.1039/c8ra03818j.

[7] Guo, H.; Yuan, D.; Fu, G. Enhanced surface imprinting of lysozyme over a new kind of magnetic chitosan submicrospheres. J. Colloid Interface Sci. 2015, 440, 53–59, https://doi.org/10.1016/j.jcis.2014.10.059.

[8] He, H.; Fu, G.; Wang, Y.; Chai, Z.; Jiang, Y.; Chen, Z. Imprinting of protein over silica nanoparticles via surface graft copolymerization using low monomer concentration. Biosens. Bioelectron. 2010, 26, 760–765, https://doi.org/10.1016/j.bios.2010.06.043.

[9] Xu, X.; Guo, P.; Luo, Z.; Ge, Y.; Zhou, Y.; Chang, R.; Du, W.; Chang, C.; Fu, Q. Preparation and characterization of surface molecularly imprinted films coated on multiwall carbon nanotubes for recognition and separation of lysozyme with high binding capacity and selectivity. RSC Adv. 2017, 7, 18765–18774, https://doi.org/10.1039/c6ra28063c.

[10] Lan, F.; Ma, S.; Ma, J.; Yang, Q.; Yi, Q.; Wu, Y.; Gu, Z. Superparamagnetic nanocomposites based on surface imprinting for biomacromolecular recognition. Mater. Sci. Eng. C 2017, 70, 1076–1080, https://doi.org/10.1016/j.msec.2016.03.048.

[11] Zhang, Z.; Wang, H.; Wang, H.; Wu, C.; Li, M.; Li, L. Fabrication and evaluation of molecularly imprinted magnetic nanoparticles for selective recognition and magnetic separation of lysozyme in human urine. Analyst 2018, 143, 5849–5856, https://doi.org/10.1039/c8an01746h.

[12] Zhang, M.; Zhang, X.; He, X.; Chen, L.; Zhang, Y. A self-assembled polydopamine film on the surface of magnetic nanoparticles for specific capture of protein. Nanoscale 2012, 4, 3141–3147, https://doi.org/10.1039/c2nr30316g.

[13] Li, N.; Qi, L.; Shen, Y.; Qiao, J.; Chen, Y. Novel Oligo(ethylene glycol)-Based Molecularly Imprinted Magnetic Nanoparticles for Thermally Modulated Capture and Release of Lysozyme. ACS Appl. Mater. Interfaces 2014, 6, 17289–17295, https://doi.org/10.1021/am505427j.

[14] Lin, Z.; Xia, Z.; Zheng, J.; Zheng, D.; Zhang, L.; Yang, H.; Chen, G. Synthesis of uniformly sized molecularly imprinted polymer-coated silica nanoparticles for selective recognition and enrichment of lysozyme. J. Mater. Chem. 2012, 22, 17914–17922, https://doi.org/10.1039/c2jm32734a.
